# Author’s Response Regarding the Relative Importance of Demographic, Socioeconomic and Health Factors on Life Expectancy in Low- and Lower-Middle-Income Countries

**DOI:** 10.2188/jea.JE20150046

**Published:** 2015-06-05

**Authors:** Md. Nazrul Islam Mondal, Mahendran Shitan

**Affiliations:** 1 Laboratory of Computational Statistics and Operations Research, Institute for Mathematical Research, University Putra Malaysia, Selangor Darul Ehsan, Malaysia; 2 Department of Mathematics, Faculty of Science, University Putra Malaysia, Selangor Darul Ehsan, Malaysia; 3 Department of Population Science and Human Resource Development, University of Rajshahi, Rajshahi, Bangladesh

Dear Prof. Inoue,

We would like to thank Dr. Selck for his interest in our article, “Relative Importance of Demographic, Socioeconomic and Health Factors on Life Expectancy in Low- and Lower-Middle-Income Countries”.^1^ We are very grateful to him for his letter^2^ having scholastic suggestions regarding our study. In that study, an attempt was made to identify the pathways by which demographic changes, socioeconomic inequalities, and availability of health factors influence life expectancy in low- and lower-middle-income countries (*n* = 91). The response variable was life expectancy, and the explanatory variables were demographic events (total fertility rate and adolescent fertility rate), socioeconomic status (mean years of schooling and gross national income per capita), and health factors (physician density and human immunodeficiency virus [HIV] prevalence). Path analysis was used to determine the direct, indirect, and total effects of these factors on life expectancy. Variables that had the most significant effects on life expectancy in the most recent previous studies were chosen.^3^^–^^6^ However, in his letter,^2^ Dr. Selck claimed that we failed to either reference or test for the assertion that, “the current increases in life expectancy have been attributed to improvements in sanitation.” Actually, the complete sentence was: “Increases in life expectancy have been attributed to improvements in sanitation and access to clean water; medical advances, including childhood vaccines; and massive increases in agricultural production.” The preceding sentence was “Wide variations in life expectancy still exist between high- and low-income countries” which demanded the next sentence to clarify the stipulation of this study. However, we do agree with the suggestion and believe a reference is merited. Further, Selck^2^ stated that the analysis of that study^1^ included recent data for poor- and middle-income countries that only focused on fertility, schooling, income, physician density, and HIV prevalence. Selck^2^ subsequently recommended that ‘sanitation’ and other factors (eg, vaccination) be included in the multivariate regression model to improve results. The suggestion is good, and we do believe that the inclusion of sanitation and vaccination as determinant factors would make the paper more relevant. We hope to incorporate the suggested factors in our next paper.
